# Prophylactic percutaneous intra-aortic balloon occlusion reduces blood loss during anterior acetabular fracture surgery: a propensity score-matched study

**DOI:** 10.1007/s00402-026-06329-w

**Published:** 2026-04-26

**Authors:** Shozo Kanezaki, Masashi Miyazaki, Akihiro Hino, Masahiro Kawagishi, Jun Nishine, Takuto Shigemi, Nobuhiro Kaku

**Affiliations:** 1 Department of Orthopaedic Surgery, Oita University, Oita, Japan; 2 Advanced Trauma and Critical Care Center, Oita University Hospital, Oita, Japan

**Keywords:** IABO (intra-aortic balloon occlusion), REBOA (resuscitative endovascular balloon occlusion of the aorta), Acetabular fracture, Anterior approach, Blood loss

## Abstract

**Introduction:**

Intra-aortic balloon occlusion (IABO) is used to control pelvic hemorrhage and has recently been adapted to elective surgery to limit intraoperative blood loss. Its role in acetabular fracture fixation, particularly via anterior approaches, remains unclear. This study evaluated whether prophylactic percutaneous IABO is associated with reduced blood loss during anterior open reduction and internal fixation (ORIF) of acetabular fractures, using propensity score matching (PSM) to minimize selection bias.

**Materials and methods:**

We conducted a retrospective cohort study including 80 consecutive adult patients who underwent anterior ORIF for acetabular fractures between 2013 and 2024. Twenty-four patients received prophylactic percutaneous IABO, and 56 served as controls. One-to-one PSM (caliper 0.2 SD of the logit) was performed on demographics, fracture characteristics, and surgical factors, yielding 20 matched pairs. Outcomes included intraoperative blood loss (IBL; primary), total blood loss (TBL; gross formula), operative time, transfusion, reduction quality, perioperative complications, and IABO-related parameters.

**Results:**

IABO was associated with significantly lower blood loss both before and after matching. In matched pairs, median IBL was 525 g versus 1070 g in controls (*p* = 0.004), representing a 51% reduction, while TBL was 601 g versus 921 g (*p* = 0.003), corresponding to a 35% reduction. Operative time, reduction quality, infection, and venous thromboembolism did not differ between groups. In the IABO cohort, the median insertion time was 13 min and balloon inflation duration was 43 min. No IABO-related complications were observed in this cohort.

**Conclusions:**

Prophylactic percutaneous IABO was associated with a significant reduction in intraoperative and total blood loss during anterior acetabular fracture fixation. Given its minimal additional setup time, it may serve as a practical adjunct in selected high-risk anterior approaches. Further prospective studies are warranted to confirm these findings.

## Introduction

Open reduction and internal fixation (ORIF) for acetabular fractures presents a considerable technical challenge due to the deep anatomical location of the acetabulum [1–3]. Intraoperative blood loss during ORIF for acetabular fractures has been reported to be considerable, particularly when an anterior approach is selected [4–7]. Managing hemorrhage during ORIF of these fractures is critical for achieving accurate and proper fracture reduction and fixation [8], that is essential for favorable clinical outcomes. Furthermore, excessive intraoperative bleeding even poses a serious threat to the patient’s life. Although various methods to reduce intraoperative blood loss in acetabular fractures have been reported, e.g., tranexamic acid, hypotensive anesthesia, and timing of operative treatment, none have demonstrated a substantial impact [5, 9, 10].

Intra-aortic balloon occlusion (IABO), also known as resuscitative endovascular balloon occlusion of the aorta (REBOA), has gained increasing recognition as an effective technique for managing hemorrhagic shock caused by torso injuries by temporarily restricting thoracic or abdominal aortic blood flow [11–13]. It has demonstrated particular value in pelvic trauma, where rapid deployment of IABO improves hemodynamic stability and survival outcomes [14–16].

More recently, IABO has also been adapted in elective surgeries to control intraoperative bleeding in high-risk settings, such as cesarean hysterectomy [17] and pelvic tumor resection [18]. Although the utility of IABO has been explored in trauma and oncologic surgeries, its clinical efficacy and safety in acetabular fracture fixation—particularly through anterior approaches that require deep anatomical exposure and are associated with significant bleeding—have not been systematically evaluated and remain to be fully elucidated [19, 20]. The present study, therefore, aims to assess the effectiveness of prophylactic percutaneous IABO in reducing intraoperative blood loss during ORIF for acetabular fractures performed through anterior approaches.

## Materials and methods

### Study design and setting

This retrospective case–control study was conducted at a single academic teaching hospital in Japan. Patient data were extracted from institutional electronic medical records and entered into standardized case report forms. We identified all consecutive patients who underwent open reduction and internal fixation (ORIF) for acetabular fractures via an anterior approach between January 2013 and December 2024.

This study was approved by the institutional ethics committee and conducted in accordance with its guidelines and the 1964 Declaration of Helsinki and its subsequent amendments. As a retrospective and non-invasive study, informed consent was obtained through an opt-out process, and the need for written informed consent was waived by the ethics committee.

### Participants

Patients aged ≥ 18 years who underwent ORIF for acetabular fractures via an anterior approach were eligible. Exclusion criteria included:

- delayed surgery (> 3 weeks post-injury),
- acute total hip arthroplasty at the index procedure, and.
- bilateral acetabular fractures.

The final study cohort included 80 patients: 24 in the IABO group and 56 in the control group. IABO has been used at our institution since 2021. Initially, IABO was selectively employed in cases considered to be at high risk for intraoperative bleeding based on the surgeon’s judgment, such as associated fracture patterns or patients with a large body habitus. In more recent practice, it has been used routinely for anterior approaches, except in patients with severe arteriosclerosis.

### Surgical procedure

**Fig. 1:**
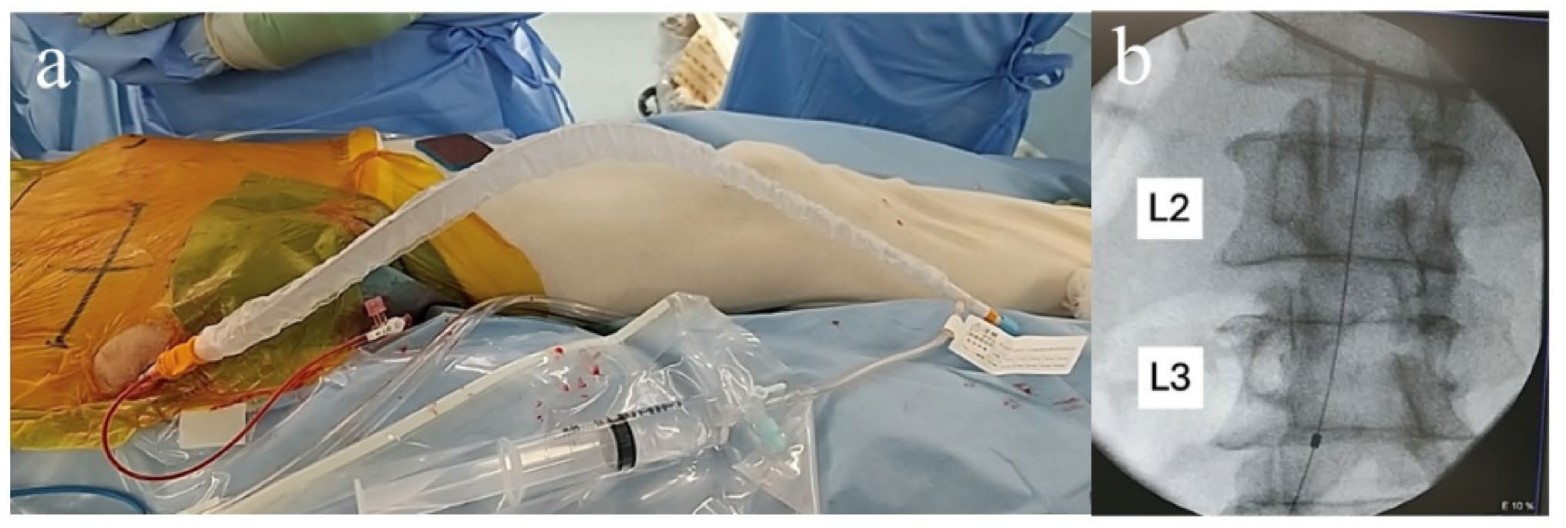
a Percutaneous insertion of the intra-aortic balloon catheter via the contralateral femoral artery under ultrasound guidance. **b** Fluoroscopic image showing the tip of the intra-aortic balloon catheter positioned in Zone 3 of the aorta, between the renal arteries and the aortic bifurcation

After induction of general anesthesia, patients were positioned supine. A bump was placed under the ipsilateral hip when a lateral window was required. In patients undergoing IABO, the device (RESCUE BALLOON-ER, East Medical Products) was inserted percutaneously via the contralateral femoral artery under ultrasound guidance, prior to surgical exposure (Fig. 1a).

The balloon catheter was advanced through a 7-Fr low-profile sheath under fluoroscopic guidance to Zone 3 of the aorta, located between the renal arteries and the aortic bifurcation. The balloon volume (6–10 mL of saline) was titrated until the contralateral femoral pulse disappeared, confirming adequate occlusion, after which the balloon was deflated. Balloon position was preoperatively planned using CT imaging to confirm the anatomical location of Zone 3, which typically lies at the level of the L2–L3 vertebral bodies (Fig. 1b).

Fracture exposure was achieved using either the anterior intrapelvic (modified Stoppa) approach or the pararectus approach. In cases where significant bleeding was anticipated, the balloon was re-inflated intraoperatively prior to exposure of the fracture site or the quadrilateral surface. Each balloon inflation was limited to a maximum of 40 min, with a minimum deflation interval of 10 min between re-inflations, if required. Following fracture reduction and fixation, hemostasis was confirmed, and routine wound closure was performed.

The balloon catheter was removed after completion of the procedure. Manual compression of the femoral puncture site was maintained for 15 min until extubation, followed by application of a pressure band, which was kept in place for 6 h postoperatively.

### Propensity score matching

To reduce confounding by indication, 1:1 propensity score matching was performed. Propensity scores for IABO use were estimated using logistic regression including the following variables:

- age group (18–44, 45–74, ≥ 75 years),
- sex,
- associated fracture type,
- low-energy trauma mechanism,
- body mass index (BMI),
- use of the lateral window, and.
- time from injury to surgery.

Nearest-neighbor matching without replacement was conducted using a caliper width of 0.2 of the standard deviation of the logit of the propensity score. Covariate balance before and after matching was assessed using standardized mean differences (SMDs), with values < 0.20 considered to indicate adequate balance. SMDs are commonly used in observational studies as a scale-invariant metric to quantify group imbalance.

### Outcome measures

1. Patient Characteristics.
  Demographic and injury-related variables extracted from the medical records included age, sex, fracture type (elementary or associated according to the Letournel classification), injury mechanism (low- or high-energy), BMI, and time from injury to surgery.
2. Primary Outcome.
  The primary outcome was intraoperative blood loss (IBL), measured directly during surgery. IBL was calculated as the suction canister volume minus irrigation fluid plus the estimated blood absorbed by surgical sponges based on weight differences. A cell-saver system was not used in these procedures.
3. Key Secondary Outcome.
  The key secondary outcome was total blood loss (TBL), calculated using the Gross formula based on changes in hematocrit values and estimated patient blood volume (PBV), as proposed by Nadler et al. [21]:
$${\mathrm{PBV}}={{\mathrm{k}}_{\mathrm{1}}} \times {\mathrm{heigh}}{{\mathrm{t}}^{\mathrm{3}}}{\text{ (m)}}+{{\mathrm{k}}_{\mathrm{2}}} \times {\text{weight (kg}})+{{\mathrm{k}}_{\mathrm{3}}},$$
  where:
  for men: k₁ = 0.3669, k₂ = 0.03219, k₃ = 0.6041.
  for women: k₁ = 0.3561, k₂ = 0.03308, k₃ = 0.1833.
  TBL = PBV × (Hct_pre − Hct_post) / Hct_ave.
  Hct_pre = preoperative hematocrit.
  Hct_post = postoperative hematocrit (Day 1).
  Hct_ave = average of Hct_pre and Hct_post.
  If allogeneic transfusion was administered, TBL was calculated as the sum of hematocrit-based estimated blood loss and transfused blood volume.
4. Other Secondary Outcomes.
  Secondary outcomes included operative time, transfusion volume and requirements, postoperative reduction quality (assessed via radiographs), and complications (infection and venous thromboembolism).
  Transfusion was generally considered when hemoglobin levels fell below 8 g/dL or when clinically indicated based on hemodynamic status.
  In patients receiving IABO, we also recorded:
  time required for IABO insertion,
  duration of balloon inflation (calculated as the cumulative duration when multiple inflations were performed),
  change in systolic blood pressure after inflation and deflation,
  and any complications directly related to the IABO procedure.

### Statistical analysis

Continuous variables were reported as median with interquartile range (IQR), and categorical variables as counts (percentages). The Mann–Whitney U test was used for continuous variables, and Fisher’s exact test for categorical variables. All statistical tests were two-tailed, with significance set at *p* < 0.05. Analyses were performed using SPSS v28 (SPSS Inc., Chicago, IL, USA).

To assess potential temporal confounding, an additional analysis was performed within the non-IABO cohort by dividing patients into three chronological periods according to the order of surgery. Perioperative outcomes were compared across these periods using the Kruskal–Wallis test.

## Results

### Patient characteristics

A total of 80 patients were included in the analysis: 24 in the IABO group and 56 in the non-IABO group. Prior to propensity score matching, notable imbalances were observed in baseline characteristics, particularly in sex distribution and the use of the lateral window (Table 1). After 1:1 propensity score matching, 20 matched pairs were generated. Standardized mean differences for all covariates were reduced to < 0.2, indicating acceptable covariate balance between the two groups (Table 2).

**Table 1:** Patient demographics and injury characteristics before propensity score matching

Variable	IABO group (*n* = 24)	Non-IABO group (*n* = 56)	*p* value	SMD
Age, median [IQR] (years)	63 [49–73]	68 [56–75]	0.332	0.193
Sex (male: female)	22:02	42:14	0.128	0.416
Fracture type (simple: associated)	8:16	21:35	0.803	0.087
Lateral window, n	12	39	0.128	0.409
Days to surgery, median [IQR]	4.0 [3.0–5.0]	4.0 [3.0–5.8]	0.174	0.425
BMI, median [IQR]	23.7 [21.4–26.0]	22.9 [20.1–25.0]	0.379	0.178
Isolated anterior column fracture, n	5	17	0.428	0.213
Dome impaction fracture, n	7	16	1	0.013
Combined pelvic ring injury, n	0	6	0.171	0.407
Low-energy trauma, n	4	8	0.746	0.067

**Table 2:** Patient demographics and injury characteristics after propensity score matching

Variable	IABO group (*n* = 20)	Non-IABO group (*n* = 20)	*p* value	SMD
Age, median [IQR] (years)	63 [47–72]	67 [56–72]	0.624	0.161
Sex (male: female)	18:02	17:03	1	0.151
Fracture type (simple: associated)	7:13	7:13	1	0
Lateral window, n	12	12	1	0
Days to surgery, median [IQR]	4.0 [3.0–5.0]	4.0 [3.0–5.8]	0.96	0.12
BMI, median [IQR]	23.2 [21.2–25.0]	23.0 [21.2–25.9]	0.96	0.081
Isolated anterior column fracture, n	4	6	0.716	0.23
Dome impaction fracture, n	7	6	1	0.107
Combined pelvic ring injury, n	0	0	1	0
Low-energy trauma, n	4	0	0.106	0.067

### Primary outcome: intraoperative blood loss

In the unmatched cohort, median intraoperative blood loss (IBL) was significantly lower in the IABO group compared to the non-IABO group (440 g [IQR, 343–735] vs. 1090 g [663–1522]; *p* < 0.0001) (Table 3). This difference remained statistically significant after propensity score matching (525 g [348–735] vs. 1070 g [555–1522]; *p* = 0.004) (Table 4).

**Table 3:** Perioperative outcomes before propensity score matching

Variable	IABO group (*n* = 24)	Non-IABO group (*n* = 56)	*p* value
Intraoperative blood loss (g), median [IQR]	440 [343–735]	1090 [663–1522]	< 0.0001
Intraoperative autotransfusion (g), median [IQR]	0 [0–0]	104 [0–374]	< 0.0001
Patients requiring autotransfusion, n	2	35	< 0.0001
Intraoperative RBC transfusion (ml), median [IQR]	0 [0–490]	420 [0–770]	0.044
Postoperative RBC transfusion (ml), median [IQR]	0 [0–0]	0 [0–0]	0.259
Patients requiring RBC transfusion, n	11	37	0.135
Drain output (g), median [IQR]	180 [110–288]	205 [150–285]	0.582
Total blood loss (g), median [IQR]	598 [355–774]	983 [654–1484]	0.0004
Hidden blood loss (g), median [IQR]	–128 [–321–45]	–193 [–761–17]	0.322
Surgical time (min), median [IQR]	189 [166–240]	225 [185–280]	0.009
Quality of reduction (anatomical/satisfactory/unsatisfactory)	16/7/1	29/24/3	0.303
Venous thromboembolism, n	1	3	1
Infection, n	1	2	1

**Table 4:** Perioperative outcomes after propensity score matching

Variable	IABO group (*n* = 20)	Non-IABO group (*n* = 20)	*p* value
Intraoperative blood loss (g), median [IQR]	525 [348–735]	1070 [555–1522]	0.004
Intraoperative autotransfusion (g), median [IQR]	0 [0–0]	104 [0–374]	0.021
Patients requiring autotransfusion, n	1	10	0.003
Intraoperative RBC transfusion (ml), median [IQR]	280 [0–490]	280 [0–770]	0.352
Postoperative RBC transfusion (ml), median [IQR]	0 [0–0]	0 [0–0]	0.423
Patients requiring RBC transfusion, n	11	12	1
Drain output (g), median [IQR]	195 [118–305]	215 [171–265]	0.795
Total blood loss (g), median [IQR]	601 [405–744]	921 [730–1544]	0.003
Hidden blood loss (g), median [IQR]	–143 [–364–45]	–100 [–426–104]	0.968
Surgical time (min), median [IQR]	203 [174–254]	215 [178–269]	0.562
Quality of reduction (anatomical/satisfactory/unsatisfactory)	13/6/1	12/7/1	0.810
Venous thromboembolism, n	1	1	1
Infection, n	1	0	1

### Key secondary outcome: total blood loss

Total blood loss (TBL), estimated using the Gross formula, was also significantly lower in the IABO group. In the unmatched cohort, median TBL was 598 g [355–774] in the IABO group and 983 g [654–1484] in the non-IABO group (*p* = 0.0004) (Table 3). After matching, the IABO group continued to show a significantly lower TBL (601 g [405–744] vs. 921 g [730–1544]; *p* = 0.003) (Table 4).

### Other secondary outcomes

No significant differences were observed between the groups in other perioperative outcomes, including operative time, intraoperative autotransfusion, allogeneic transfusion requirements, postoperative reduction quality, and the incidence of complications such as infection or venous thromboembolism (Tables 3 and 4).

In the IABO group, the median time required for device insertion was 13 min [IQR, 10–16]. The median duration of balloon inflation was 43 min [37–73]. Systolic blood pressure increased by a median of 14 mmHg [5–19] after balloon inflation and decreased by 30 mmHg [13–37] following deflation. No IABO-related complications were observed in any case.

### Temporal analysis of the non-IABO cohort

The non-IABO cohort was subdivided into three chronological periods (19, 19, and 18 cases) to assess potential temporal confounding. No significant differences were observed in intraoperative blood loss, total blood loss, hidden blood loss, transfusion volume, or postoperative drain output across the three periods. The highest intraoperative blood loss was observed in the intermediate period rather than in the earliest period. Operative time decreased significantly across periods (Table 5).

**Table 5:** Temporal analysis of perioperative outcomes in the non-IABO cohort

Variable	Period 1 (*n* = 19)	Period 2 (*n* = 19)	Period 3 (*n* = 18)	*p* value
Age (years)	68	66	71	0.084
Operative time (min)	269.5	214.5	188.5	< 0.001
Intraoperative blood loss (g)	1100	1445	760	0.254
Total blood loss (g)	1214	1112	742	0.085
Hidden blood loss (g)	−180	−87	−317	0.617
Intraoperative RBC transfusion (ml)	280	560	280	0.555
Postoperative RBC transfusion (ml)	0	0	0	0.146
Drain output POD1 (g)	210	200	205	0.999

## Discussion

This study demonstrated that prophylactic use of intra-aortic balloon occlusion (IABO) during anterior acetabular fracture surgery was associated with a significant reduction in both intraoperative blood loss (IBL) and total blood loss (TBL), even after adjustment for baseline characteristics using propensity score matching. These findings suggest that IABO may be an effective adjunctive strategy for blood loss management in selected high-risk cases requiring anterior approaches.

Our findings align with those of previous retrospective studies [19, 20], which have also reported favorable outcomes associated with temporary aortic balloon occlusion in acetabular fracture surgery. Kong et al. [20] demonstrated in a cohort of 43 patients with delayed acetabular fractures that temporary aortic occlusion significantly reduced operative time, blood loss, and transfusion requirements, without increasing complications. Similarly, Hao et al. [19] reported comparable benefits in 48 patients with complex fractures, supporting the utility of aortic balloon occlusion as a hemostatic adjunct in high-risk pelvic procedures.

Unlike these earlier studies, which used open surgical techniques for catheter placement and did not account for confounding variables, our study employed a percutaneous approach using a 7-Fr low-profile sheath, which allows for rapid and less invasive arterial access. Moreover, we applied propensity score matching to minimize selection bias. The median catheter insertion time was 13 min, a duration considered clinically acceptable. These methodological refinements enhance the internal validity and generalizability of our findings.

Although the optimal duration of balloon inflation remains uncertain, we limited each inflation period to 40 min based on the experimental study by Avaro et al. [22], which demonstrated improved survival and reduced ischemic injury with this threshold in a hemorrhagic shock model. While serious complications—such as aortic rupture [23], arterial thrombosis [24], spinal cord ischemia [25], and renal dysfunction [26]—have been reported in other settings, no IABO-related complications were observed in this cohort. These findings are consistent with those of Teeter et al. [27], who reported favorable safety outcomes with the use of 7-Fr IABO catheters in a multicenter trauma registry. However, the sample size was insufficient to evaluate rare vascular complications. Furthermore, there were no significant differences in the incidence of symptomatic deep vein thrombosis or pulmonary embolism between the IABO and non-IABO groups.

Despite these strengths, several limitations should be acknowledged. First, this was a retrospective, single-center study, which may introduce selection and institutional biases. During the early phase after the introduction of IABO, its use was determined at the discretion of the operating surgeon for cases perceived to be at higher risk of bleeding, such as associated fracture patterns or patients with a large body habitus. This selective use may have introduced selection bias; however, such bias would likely favor the non-IABO group because IABO tended to be used in more technically demanding cases. Because IABO was introduced at our institution in 2021, the IABO group represents a more recent surgical period than the control group; therefore, temporal changes in surgical practice could theoretically influence perioperative outcomes. Additional analyses within the non-IABO cohort demonstrated no significant differences in blood-loss-related outcomes across chronological periods, suggesting that temporal improvements alone are unlikely to explain the observed association. Although propensity score matching was used to reduce confounding, residual confounding from unmeasured variables cannot be excluded. Second, the sample size after matching was relatively small, which may have limited the power to detect infrequent adverse events. Third, the study focused primarily on perioperative outcomes; long-term functional results and patient-reported outcomes were not evaluated. Fourthly, negative hidden blood loss values may occur in hematocrit-based calculations due to perioperative hemodilution and fluid administration. Therefore, total blood loss estimated using the Gross formula should be interpreted as an approximation with recognized methodological limitations. Lastly, cost-effectiveness comparisons between IABO and alternative blood-conservation strategies—such as tranexamic acid administration or hypotensive anesthesia—were beyond the scope of this study but warrant future investigation.

Despite these limitations, the consistency of findings after propensity score matching supports the robustness of the observed association. Taken together, these findings suggest that prophylactic IABO may serve as a practical and safe hemostatic adjunct in selected high-risk anterior acetabular fracture surgeries. To further establish its clinical utility, multicenter prospective studies are warranted to validate its efficacy and cost-effectiveness.

## Conclusions

Using propensity score matching to minimize selection bias, this study demonstrated that prophylactic percutaneous IABO was associated with a significant reduction in intraoperative and total blood loss during anterior acetabular fracture fixation. Given its minimal additional setup time, it may serve as a practical adjunct in selected high-risk anterior approaches. However, due to the retrospective design and potential residual confounding, these findings should be interpreted with caution. Further prospective, multicenter studies are warranted to validate its clinical utility and cost-effectiveness.

## Data Availability

The datasets used and analyzed during the current study are available from the corresponding author on reasonable request. To ensure confidentiality of data and to avoid data loss or manipulation, precautionary measures have been taken: data availability is restricted to authorized members only.
